# Mechanism of Zn^2+^ and Ca^2+^ Binding to Human S100A1

**DOI:** 10.3390/biom11121823

**Published:** 2021-12-03

**Authors:** Viktoriia E. Baksheeva, Andrei Yu. Roman, Claude Villard, François Devred, Deborah Byrne, Dahbia Yatoui, Arthur O. Zalevsky, Alisa A. Vologzhannikova, Andrey S. Sokolov, Sergei E. Permyakov, Andrey V. Golovin, Gary S. Shaw, Philipp O. Tsvetkov, Evgeni Yu. Zernii

**Affiliations:** 1 Institut de Neurophysiopathologie, INP, CNRS, Faculté des Sciences Médicales et Paramédicales, Aix-Marseille Université, 13005 Marseille, France; vbaksheeva@belozersky.msu.ru (V.E.B.); andrei.roman@ubc.ca (A.Yu.R.); claude.villard@univ-amu.fr (C.V.); francois.devred@univ-amu.fr (F.D.); yatouidahbia19@gmail.com (D.Y.); 2 Belozersky Institute of Physico-Chemical Biology, Lomonosov Moscow State University, 119992 Moscow, Russia; 3 Institute of Physiologically Active Compounds, Russian Academy of Sciences, 142432 Chernogolovka, Russia; 4 Institut de Microbiologie de la Méditerranée, CNRS, FR3479, Aix-Marseille Université, 13402 Marseille, France; Deborah.byrne@univ-amu.fr; 5 Shemyakin-Ovchinnikov Institute of Bioorganic Chemistry, Russian Academy of Sciences, 117997 Moscow, Russia; aozalevsky@fbb.msu.ru (A.O.Z.); golovin@fbb.msu.ru (A.V.G.); 6 Institute for Biological Instrumentation, Pushchino Scientific Center for Biological Research of the Russian Academy of Sciences, 142290 Pushchino, Russia; lisiks.av@gmail.com (A.A.V.); 212sok@gmail.com (A.S.S.); permyakov.s@gmail.com (S.E.P.); 7 Faculty of Bioengineering and Bioinformatics, Lomonosov Moscow State University, 119991 Moscow, Russia; 8 Sirius University of Science and Technology, 354340 Sochi, Russia; 9 Department of Biochemistry, Schulich School of Medicine & Dentistry, University of Western Ontario, London, ON N6A 3K7, Canada; gshaw1@uwo.ca

**Keywords:** S100A1, zinc, calcium, ESI-MS, ITC, nanoDSF

## Abstract

S100A1 is a member of the S100 family of small ubiquitous Ca^2+^-binding proteins, which participates in the regulation of cell differentiation, motility, and survival. It exists as homo- or heterodimers. S100A1 has also been shown to bind Zn^2+^, but the molecular mechanisms of this binding are not yet known. In this work, using ESI-MS and ITC, we demonstrate that S100A1 can coordinate 4 zinc ions per monomer, with two high affinity (K_D_~4 and 770 nm) and two low affinity sites. Using competitive binding experiments between Ca^2+^ and Zn^2+^ and QM/MM molecular modeling we conclude that Zn^2+^ high affinity sites are located in the EF-hand motifs of S100A1. In addition, two lower affinity sites can bind Zn^2+^ even when the EF-hands are saturated by Ca^2+^, resulting in a 2Ca^2+^:S100A1:2Zn^2+^ conformer. Finally, we show that, in contrast to calcium, an excess of Zn^2+^ produces a destabilizing effect on S100A1 structure and leads to its aggregation. We also determined a higher affinity to Ca^2+^ (K_D_~0.16 and 24 μm) than was previously reported for S100A1, which would allow this protein to function as a Ca^2+^/Zn^2+^-sensor both inside and outside cells, participating in diverse signaling pathways under normal and pathological conditions.

## 1. Introduction

The S100 protein family encompasses small (~10 kDa) ubiquitous two-EF-hand Ca^2+^-binding proteins, normally existing as homo- or heterodimers and acting as both secreted and intracellular signaling molecules [1]. In cells, the S100 proteins participate in the regulation of mobility, differentiation and survival through interactions with numerous targets, such as effector enzymes, cytoskeletal proteins, receptors, and transcription factors. When released from cells, they display cytokine-like activity and bind to cell surface receptors (like toll-like receptors and G-proten-coupled receptors) or interact with growth factors, thereby regulating the activity of different immune cells, chondrocytes, myoblasts, cardiomyocytes, epitheliocytes, astrocytes, glial cells and neurons (reviewed in [2]). Abnormalities in the expression of S100 proteins are found in a number of cancers [3]. Altered expression of brain-specific S100 proteins is associated with decreased motor and cognitive skills and neurological damage characteristic of Alzheimer’s disease (AD) and Parkinson’s disease, and several members of the S100 family are found in the amyloid plaques and cerebrospinal fluid of AD patients [4,5,6,7]. Secreted S100 proteins are commonly found in the aqueous humor of patients with glaucoma, which is accompanied by overexpression of these proteins in the damaged retina [8,9]. Despite extensive knowledge of the cellular and tissue functions of S100 proteins, understanding of the exact mechanisms underlying their signaling activity remains scarce, especially regarding Ca^2+^-sensitivity and interrelations between multiple isoforms of these proteins.

Typical representatives of S100 proteins are S100A1 and S100B, expressed in the skeletal muscles and heart, as well as in the neurons, astrocytes and microglia, where they exist as homo- and heterodimers [10,11,12]. In the absence of a target, both of these proteins exhibit very low calcium affinity: K_D_ exceeds 10 μM, which is outside the intracellular concentration range for this cation [13]. There are several possible explanations for how these proteins can sense alterations in physiological concentrations of calcium. One common hypothesis is that the affinity of their Ca^2+^-coordinating sites can be adjusted via allosteric regulation provided by target binding [14]. Consistently, in the presence of one such target, the ryanodine receptor, S100A1 exhibits 100-fold increased Ca^2+^ affinity [15]. Another option is the binding of Zn^2+^, which was suggested to increase the Ca^2+^-affinity of certain S100 proteins. In recent years, Zn^2+^ has been recognized as the second major signaling ion in the nervous system (in addition to Ca^2+^) [16]. It regulates the normal functioning of the neurons and participates in the pathogenesis of neurological and neuro-ophthalmological disorders, predominantly AD and glaucoma [17,18,19]. For instance, Zn^2+^ can act as a signaling ion in the nervous system by binding to Ca^2+^-sensor proteins and modifying their structural and functional properties, such as Ca^2+^-sensitivity, affinity to cellular membranes or target proteins, and a propensity for non-covalent/covalent (disulfide) dimerization and aggregation [20,21,22]. In the S100B homodimer, the binding of Zn^2+^ greatly improves the Ca^2+^-affinity of the protein, promoting its activation by Ca^2+^ signals and the recognition of regulatory targets [13,23,24,25]. At the same time, Zn^2+^ has been reported to have no such effect on S100B/S100A1 heterodimers, which leads to the suggestion that S100A1 serves as an inhibitor of the Zn^2+^-dependent function of S100B [13]. S100A1 is also able to function independently from S100B, as it forms homodimers and can directly bind Ca^2+^ and Zn^2+^ [13]. Notably, previous studies have shown that the metal-binding properties of S100A1 drastically differ from those of S100B, pointing to its unique mode of its function. 

In this study, using electrospray mass-spectrometry, isothermal titration calorimetry (ITC), dynamic light scattering (DLS), differential scanning fluorimetry (nanoDSF), and QM/MM molecular modeling, we investigated the mechanisms of Ca^2+^ and Zn^2+^ coordination in S100A1, including the structure and interrelations of its metal-binding sites. Our findings suggest that S100A1 can function as a Ca^2+^/Zn^2+^-sensor protein, mediating the fluctuations in both ions as a part of the diverse signaling pathways in the nervous system. Moreover, we demonstrate that an excess of Zn^2+^ produces a destabilizing effect on S100A1 structure and leads to the aggregation of the protein, which could contribute to its neurotoxicity under the pathological conditions of glaucoma and AD.

## 2. Materials and Methods

### 2.1. Protein Purification

The recombinant S100A1 protein was purified from *Escherichia coli* culture, as described previously [26]. Briefly, the bacterial extract was subjected to two steps of chromatography: anion exchange chromatography on a HiTrap Q column (GE Healthcare, Chicago, IL, USA), using a linear gradient of 0–1 M NaCl; and Ca^2+^-dependent affinity chromatography on a phenyl-Sepharose column (GE Healthcare), where the S100A1-containing fractions from the first step were pooled and loaded on the column in 5 mM CaCl_2_, and the pure protein was eluted with 5 mM ethylene glycol-bis(β-aminoethyl ether)-N,N,N′,N′-tetraacetic acid (EGTA) (Sigma-Aldrich, Saint-Loius, MO, USA). Residual Ca^2+^ was removed from the protein according to the previously described procedure [20,27]. For circular dichroism (CD) studies (see below), recombinant S100A1 was obtained as described previously [28].

### 2.2. Electrospray Mass-Spectrometry (ESI-MS)

Mass spectrometric measurements of 15 µM of S100A1 in the presence of 200 µM of zinc or calcium ions were performed on an LCQ Deca XP ion trap mass spectrometer (Thermo Finnigan, San-Jose, CA, USA) equipped with an electrospray ionization source, as described previously [29]. All experiments were performed in a 10 mM ammonium bicarbonate buffer (pH 7.0). The infusion flow rate was 0.5 µL/min, the spray voltage was 4 kV, and the nitrogen sheath gas setting was 20 au. Full scans of m/z 1500–4000 were acquired using the Excalibur 1.3 data system (Thermo Finnigan, San-Jose, CA, USA). 

### 2.3. Isothermal Titration Calorimetry

The binding of ions to the S100A1 protein was studied on an iTC200 isothermal titration calorimeter (MicroCal, Los Angeles, CA, USA) at 37 °C, in 50 mM Tris buffer and 1 mM tris(2-carboxyethyl)phosphine (TCEP) at pH 7.5, as described previously [20,30]. The protein concentration in the calorimetric cell was 50 µM whereas the concentration of zinc and calcium ions in the titrating syringe was 0.5 mM. The volume of the injections was 0.5 µL before the ion concentration reached the equimolar ratio with protein, and then set to 2.5 µL. The dilution heat was measured by titration of the ion-containing buffer into the same buffer without the protein. The obtained curve was analyzed by MicroCal Origin 7.0 software (Los Angeles, CA, USA) using the models of the two types of binding sites. Therefore, the stoichiometry (N), constant (K_a_), enthalpy (ΔH), and entropy of binding (ΔS) were calculated from the standard thermodynamic equations.

### 2.4. Dynamic Light Scattering (DLS)

All DLS experiments were carried out at 37 °C using a Zetasizer Nano ZS (Malvern Instruments, Malvern, UK) as described previously [31]. S100A1 size was determined in the presence of 0.5–10 mM of Zn^2+^ or Ca^2+^ or in the absence of divalent metal ions. The protein concentration was 50 µM. The hydrodynamic diameter was determined using the software provided by the manufacturer. The dispersant viscosity was set to 0.6909 cP and the protein refractive index was set to 1.450 (at 37 °C). Several replicate measurements were performed; each one consisting of 10–15 runs of 10 s.

### 2.5. Differential Scanning Fluorimetry (nanoDSF) and Light Scattering 

Protein thermostability was estimated using a label-free fluorimetric analysis in a Prometheus NT.Plex instrument (NanoTemper Technologies, Munich, Germany) as described in our previous works [20,31]. A total of 37 µM of S100A1 (the concentration corresponds to the protein concentration in a calorimetric cell at the end of ITC titration) was loaded into NanoDSF grade capillaries (NanoTemper Technologies, Munich, Germany) in the presence of either 1 mM Ca^2+^, 1 mM Zn^2+^, or neither. The concentration of zinc or calcium ions varied from 18.5 to 296 µM (excesses 0.5 to 8). The capillaries were loaded onto the instrument and heated from 20 °C to 110 °C at a 1 K/min heating rate with the excitation laser power set at 100%. The unfolding transition points (T_m_) were determined from the first derivative of the changes in the emission wavelengths of ratio of tryptophan fluorescence intensities at 350 nm and 330 nm (I_350_/I_330_), which were automatically identified by the pre-installed PR.ThermControl software (NanoTemper Technologies, Munich, Germany). In addition, the aggregation temperatures (T_agg_) were determined based on first derivative of temperature dependence of light scattering at 350 nm, using the same equipment. 

### 2.6. Circular Dichroism

Circular dichroism (CD) studies were carried out with a J-810 spectropolarimeter JASCO Inc., Tokyo, Japan), equipped with a Peltier-controlled cell holder, as previously described [32]. Protein concentration was 34–37 µM in 50 mM Tris-HCl, 1 mM TCEP pH 7.5. A total of 1 mM CaCl_2_ (for Ca^2+^-loaded form) or 1 mM ZnCl_2_ (for Zn^2+^-loaded form) was added to the metal-depleted protein and pre-incubated for 5 min. A total of 296 µM ZnCl_2_ (Zn^2+^:protein ratio 8:1) was added to the Ca^2+^-loaded form or 296 µM CaCl_2_ (Ca^2+^:protein ratio 8:1) was added to the Zn^2+^-loaded form. The buffer contribution was subtracted from the experimental spectra. Quantitative estimates of the secondary structure contents were made using the CDPro software package (Colorado State University, Fort Collins, CO, USA) [33]. The finalized secondary structure fractions represent the averaged values from several measurements.

### 2.7. QM/MM Molecular Modeling

Quantum mechanics/molecular mechanics (QM/MM) simulations using the GROMACS-DFTb/Plumed package (https://www.gromacs.org/) were performed as described in [20,34]. The starting conformation of the S100A1 protein was obtained from the NMR solution structure (PDB 2LP3). Ca^2+^ was replaced with Zn^2+^ in two EF-hand sites of chain A. All residues harboring oxygen atoms in proximity to Zn^2+^ were included in the QM part of the system. In the case of oxygen atoms originating from the sidechain, QM/MM partitioning was performed on the CA-CB bond. For the backbone oxygens, partitioning was performed on the CA-C and CA-N bonds. The simulation system was filled with water molecules (presented by the tip3p model), and Na^+^ or Cl^−^ ions to neutralize the charge. The water and ions were equilibrated around the protein-Zn^2+^ complex by carrying out a 2ns MD simulation with a restrained position of protein. All atoms (including water) closer to Zn^2+^ than 3 Å were also included in the QM system. The total length of each simulation was set to 100 ps with a 0.2 fs timestep.

## 3. Results

### 3.1. Stoichiometry of S100A1-Metal Binding 

**Figure 1:**
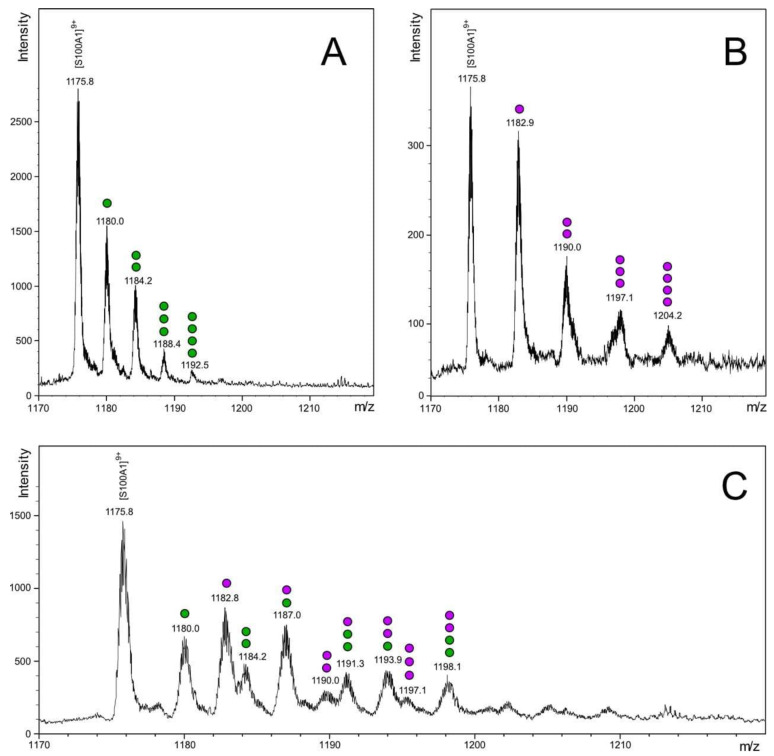
Mass-spectra of Ca^2+^- and Zn^2+^-bound S100A1. Samples containing 15 µM of S100A1 preincubated with (**A**) 200 µM Ca^2+^, (**B**) 200 µM Zn^2+^ or (**C**) both, were analyzed with electrospray mass spectrometry. Calcium ions are shown as green circles and zinc ions are shown as purple circles.

The stoichiometry of Ca^2+^ and Zn^2+^ binding to S100A1 was first analyzed by electrospray ionization mass-spectrometry (ESI-MS), as this method allows the direct monitoring of complexes of proteins with different metals. To optimize the detection of S100A1 complexes with cations, mass spectrometry experiments were carried out at pH 7.0 in the presence of 200 µM Ca^2+^ or 200 µM Zn^2+^, or both cations together [13]. In all experimental conditions, the main peak in the MS spectra was observed at 1175.8 m/z, which corresponds to the eighth charge state of the S100A1 species [S100A1^+^+8H^+^]^9+^ with a calculated average mass of 10,571.2 ± 1.4 Da (Figure 1). This value differs slightly from the theoretical mass (10,545.5 Da) due to the N-formylmethionine (fMet) modification at the N-terminus of the protein, which is unlikely to affect metal binding and therefore was not taken into account in the subsequent analysis. In the presence of calcium or zinc alone, S100A1 existed as five species corresponding to apo-protein and its conformers with one, two, three or four bound cations, the content of which decreases in the order 1Me^2+^-S100A1 > 2Me^2+^-S100A1 ≫ 3Me^2+^-S100A1 > 4Me^2+^-S100A1 (Figure 1A,B). Although this pattern does not directly reflect the S100A1 state in solution due to the destabilizing ESI-MS-related effects, it points to the existence of high/medium-affinity and low-affinity Ca^2+^/Zn^2+^-binding sites in the protein. The preincubation of S100A1 in the presence of both Ca^2+^ and Zn^2+^ yielded multiple conformers with maximum four metal ions per protein with the predominance of single-ion and two-ion forms, including a substantial amount of a mixed Zn^2+^:S100A1:Ca^2+^ form (Figure 1C). All calcium conformers contained no more than two Ca^2+^, which are most likely coordinated in the EF-hands [35,36]. Notably, the only form of S100A1 with four ions contains two Ca^2+^ and two Zn^2+^ per monomer (2Zn^2+^:S100A1:2Ca^2+^). Given the destabilizing conditions of ESI-MS experiments and the absence of other four ion-bound variants of S100A1, this form seems to represent a relatively stable conformer of the protein.

### 3.2. Affinity of S100A1-Metal Binding

**Figure 2:**
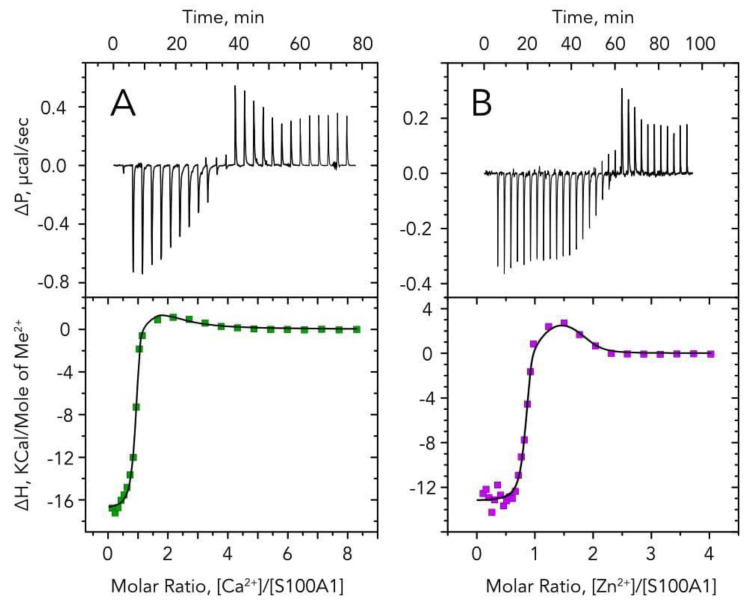
Isothermal titration calorimetry (ITC) analysis of ions binding to S100A1. A total of 50 μM of S100A1 was titrated by (**A**) 500 μM Ca^2+^ or (**B**) 500 μM Zn^2+^. Upper panels show ITC curves and lower panels show binding isotherms. Best fits are shown as solid black curves (see Table 1).

To characterize Zn^2+^ and Ca^2+^ binding to S100A1, the formation of the complexes was monitored using ITC (Figure 2) by the titration of 50 µM apo-S100A1 with 500 µM Ca^2+^ or 500 µM Zn^2+^. The fitting of the resulting titration curves using a “two sets of sites” model revealed the existence of one high- and one medium-affinity binding site for both ions (Table 1). It should be noted that both sites have two orders of magnitude higher association constants for Zn^2+^ than for Ca^2+^. For both metals, binding in the high-affinity site is enthalpy driven (ΔH < 0 and ΔS < 0), whereas binding in the medium-affinity site is entropy driven (ΔH > 0 and ΔS > 0). This indicates different conformational changes of the protein upon ion binding to either high or medium-affinity sites.

Since it is known that the two main calcium-binding sites are located in the two EF-hands of S100A1, ion competition experiments allowed us to establish whether the EF-hands of the protein can also chelate zinc ions. The presence of zinc at saturating concentrations results in the absence of heat exchange upon protein titration by calcium (and vice versa). This observation strongly suggests the existence of competition between Zn^2+^ and Ca^2+^ for both sites at S100A1, thus indicating that the Zn^2+^-binding sites are also located in the EF-hands of the protein. 

Moreover, since the binding of Ca^2+^ to its high-affinity site is stronger than the binding of Zn^2+^ to its medium-affinity site (Table 1) and since, in the presence of equimolar concentrations of both ions, the Zn^2+^:S100A1:Ca^2+^ species is much more abundant than the 2Zn^2+^:S100A1 or S100A1:2Ca^2+^ species (Figure 1C), we can conclude that high-affinity sites for these cations are localized in different EF-hands.

### 3.3. Effect of Ca^2+^ and Zn^2+^ on Secondary Structure of S100A1

Possible alterations to secondary structure induced by Ca^2+^ and Zn^2+^ binding to S100A1 were assessed by registering the circular dichroism (CD) spectra of the protein in the excess of these ions (Supplementary Materials, Figure S1). It was found that the addition of either 1 mM Ca^2+^ or 1 mM Zn^2+^ to S100A1 induces a moderate reduction to its α- and β-structures and increased its share of turns and disordered elements (Table 2). As a result, the Ca^2+^- and Zn^2+^-loaded forms of the protein exhibited similar patterns of secondary structure elements. Thus, the filling of S100A1 with calcium or zinc yielded conformers with similar secondary structures, both of which slightly differed from the apo from. 

### 3.4. Putative Zn^2+^-Binding Sites in S100A1

The results of the ESI-MS and ITC studies indicated that (1) Zn^2+^ can bind to the EF-hands of S100A1 and (2) this binding is characterized by nanomolar affinity. To assess the structural aspects underlying this high-affinity interaction, we next predicted the Zn^2+^-binding sites in the EF-hands of S100A1 in silico, based on the available NMR structure of the Ca^2+^-bound protein (PDB 2LP3 [36]), since, as shown by our CD experiments (Table 1), the Ca^2+^-bound form of the protein is similar to its Zn^2+^-bound form in secondary structure. To this end, we performed QM/MM simulations of the molecular dynamics associated with Zn^2+^ binding instead of calcium in both EF-hand sites of chain A. Prior to the simulations, the solvent components, namely water molecules and sodium and chloride ions, were added to the system to neutralize the charge and create a physiological ionic strength of 0.14 M. Although initially the structure lacked water molecules, the balancing of the positions of such molecules around the protein resulted in their embedding in both sites in the vicinity of the zinc ions. The N-terminal and C-terminal EF-hands acquired one and three water molecules, respectively. The residues of both metal-binding sites containing Zn^2+^ and the respective water molecules were included in separate QM systems and subjected to four independent molecular dynamics simulations in 500,000 steps. 

**Figure 3:**
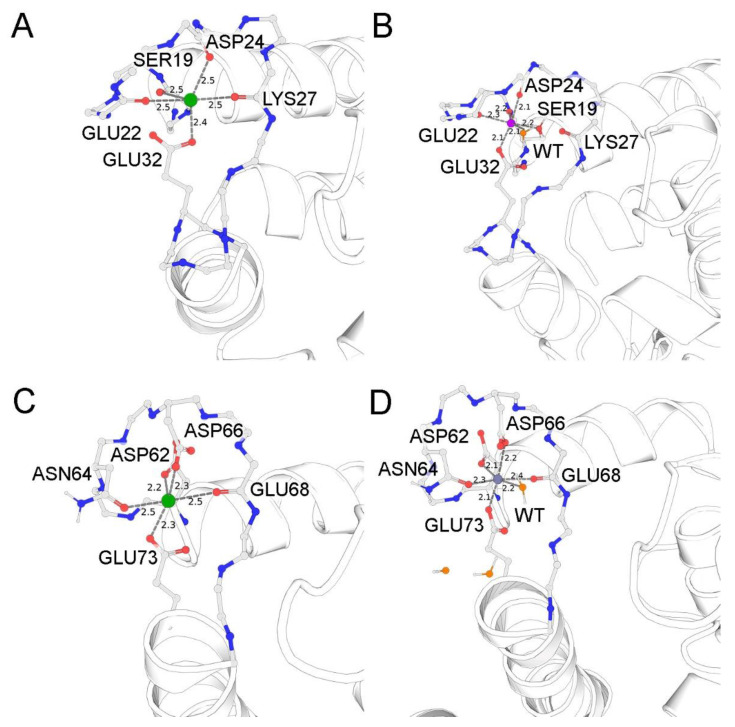
Coordination spheres of Ca^2+^ and Zn^2+^ in N-terminal (**A**,**B**) and C-terminal (**C**,**D**) EF-hands of S100A1. (**A**,**C**) NMR structure of Ca^2+^-bound S100A1 (PDB 2LP3). Calcium ions are colored green. Oxygen atoms in the coordination sphere are colored red (protein) or orange (water). The distances between chelator atoms and the metal ion are denoted in angstroms. (**B**,**D**) QM/MM models of Zn^2+^ coordination. Zinc ions are colored purple. The other designations are the same as in (**A**,**C**).

As can be seen in Figure 3A, the original coordination of calcium in the medium-affinity N-terminal EF-hand is achieved by backbone oxygens of S19, E22, D24, and K27, as well as single carboxyl oxygen of E32. The sixth coordinator might be a water molecule, which is absent in the NMR structure. In the case of zinc binding (Figure 3B), the oxygen atom of the K27 backbone is replaced by the water molecule from the corresponding coordination sphere, and the position of this molecule is stabilized by the backbone oxygen of K27 and the second carboxyl oxygen of E32. At the same time, the OG atom of S19 enters the coordination sphere, thereby creating an octahedral environment favorable for zinc binding. By contrast, in the C-terminal EF-hand, Ca^2+^ is favorably coordinated by the carboxyl groups of D62, D66, and E73 as well as the backbone oxygens of N64 and E68 (a water molecule is absent in the structure of NMR) (Figure 3C). This optimal coordination does not change during the simulation with zinc, except that the respective water molecule, stabilized by carboxyl atoms of the D66 and E73 residues completes the octahedral coordination of this metal. 

In the aggregate, our simulations suggest that the N-terminal EF-hand of S100A1 is better suited for Zn^2+^, whereas the C-terminal EF-hand seems to be a fit for both metals with a slight advantage of Ca^2+^.

### 3.5. Effects of Metal Binding on Dimeric Structure of S100A1 

**Figure 4:**
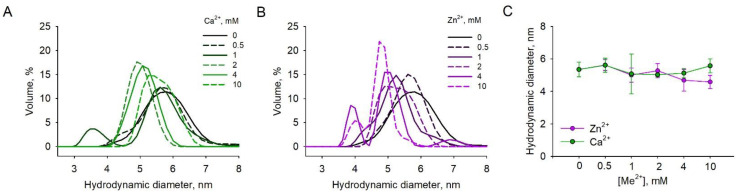
Dynamic light scattering (DLS) analysis of Ca^2+^- and Zn^2+^-loaded S100A1. A total of 50µM of S100A1 was incubated with increasing concentrations (0.5–10 mM) of (**A**) Ca^2+^ and (**B**) Zn^2+^ at 37 °C and volume distribution of S100A1 hydrodynamic diameter was determined. Lower concentrations are shown as darker coloured lines and higher concentrations are shown as lighter coloured lines. (**C**) The influence of increasing divalent ions on the major peak size of triplicate concentrations.

Given the results of the metal-binding experiments, S100A1 may exist as three main states (apo, Ca^2+^-loaded, Zn^2+^-loaded) and several mixed states (mainly Zn^2+^:S100A1:Ca^2+^ and 2Zn^2+^:S100A1:2Ca^2+^), all of which seem physiologically relevant, especially considering both the intracellular and extracellular localizations of the protein. As such, we next analyzed the multimeric structure, conformational properties and stability of these forms. The examination of the hydrodynamic properties of S100A1 by DLS revealed that in the absence of metals, its average volume diameter (Dv50) is 5.63 ± 0.422 d.nm for 6 independent measurements, confirming that the protein exists as the dimer (Figure 4). The presence of increasing concentrations of Ca^2+^ (up to 10 mM) or Zn^2+^ (up to 4 mM) does not significantly affect the average diameter of S100A1, indicating the persistence of the dimer (Figure 4A,B). The notable change in protein size, namely a decrease down to 4.6 ± 0.4 d.nm, was observed only in the presence of 10 mM Zn^2+^, apparently reflecting complete filling of all low-affinity sites of the protein and suggesting a more compacted spherical form (similar shape was observed for homologous protein S100A11 in its apo-form; PDB: 2LUC) (Figure 4C).

### 3.6. Effects of Metal Binding on Conformational Properties of S100A1

**Figure 5:**
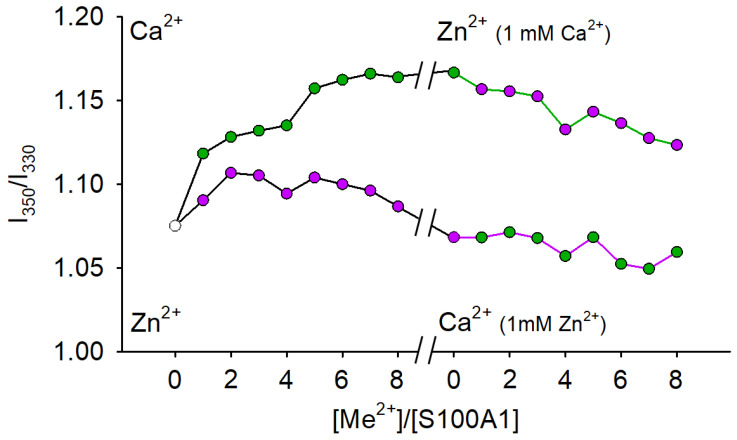
Effects of Ca^2+^ and Zn^2+^ on conformational properties of S100A1. 37 µM of S100A1 in a buffer containing Ca^2+^, Zn^2+^, or their combinations was loaded into capillaries and intrinsic tryptophan fluorescence of the protein was registered at 37 °C. The figure shows the influence of increasing concentrations of metal ions (37–296 μM) on fluorescence intensities at 350 nm and 330 nm. The titration of apo-S100A1 with Ca^2+^ is shown as green circles and black lines; titration of apo-S100A1 with Zn^2+^ is shown as purple circles with black lines; titration with Zn^2+^-saturated S100A1 with Ca^2+^ is shown as green circles with purple lines; titration of Ca^2+^-saturated S100A1 with Zn^2+^ is shown as purple circles with green lines.

To confirm the existence of the suggested states of S100A1, we analyzed its conformational changes induced by Zn^2+^ and/or Ca^2+^, by measuring fluorescence of the protein at 350 and 330 nm (I_350_ and I_330_) and 37 °C using nanoDSF. The ratio of I_350_/I_330_, which correlates with the environment of the only tryptophan (Trp90) located close to the C-terminus, was plotted as the function of the molar ratio [Me^2+^]/[S100A1] (Figure 5). The changes in the conformation of S100A1 upon the titration of its apo-form with Ca^2+^ and Zn^2+^ up to four-fold excess could be associated with metal coordination by the two EF-hand sites. Indeed, we can observe the saturation of these sites at a four-fold excess in the ITC experiments (Figure 2). The filling of the first high-affinity site is accompanied by pronounced conformational changes (molar ratio 0→1 in Figure 5), while the filling of the medium-affinity site does not significantly impact tryptophan fluorescence (molar ratio 1→4 in Figure 5). Intriguingly, the binding of Zn^2+^, despite its higher affinity, produces moderate effects on protein conformation, as seen from the smaller change in the I_350_/I_330_ ratio.

The absence of conformational changes upon the titration of the Zn^2+^-saturated protein by Ca^2+^ was in good agreement with our ITC data, thus confirming that the first two zinc ions fill and block the EF-hands. In contrast, the decrease of the I_350_/I_330_ ratio upon titration of Ca^2+^-loaded protein by Zn^2+^ points on the latter binding to Ca^2+^-loaded S100A1. At a molar ratio 4 it results in formation of a conformer that is similar to S100A1:2Ca^2+^. Apparently, this conformer contains two calcium ions in the EF-hands and 1–4 zinc ions which replaced Ca^2+^ in some nonspecific sites outside the EF-hands. Given the results of the ESI-MS experiments (see Figure 1C), this conformer most likely represents 2Zn^2+^:S100A1:2Ca^2+^. Thus, we conclude that each S100A1 monomer competitively binds two Ca^2+^ or two Zn^2+^ in the EF-hands, and can competitively bind these ions in low-affinity (nonspecific) sites outside the EF-hands, which display a preference for Zn^2+^.

### 3.7. Effects of Metal Binding on Thermal Stability and Aggregation of S100A1

**Figure 6:**
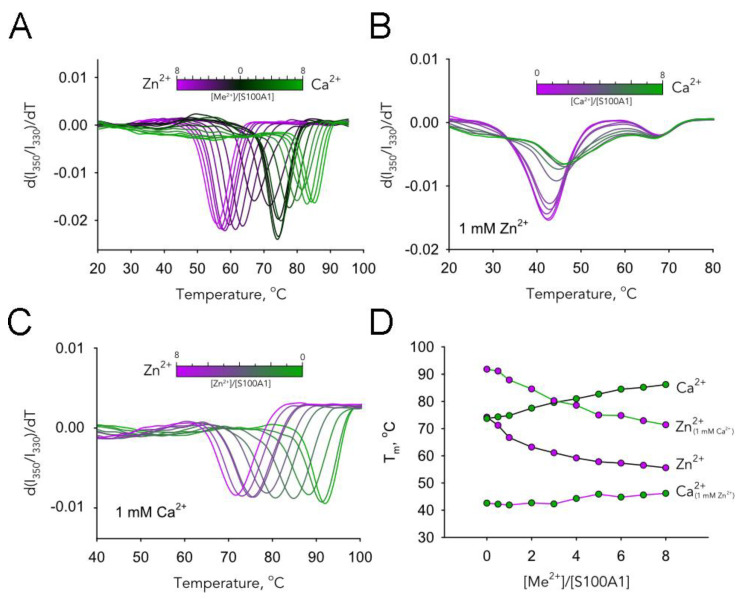
Effect of Ca^2+^ and Zn^2+^ on the thermal stability of S100A1. A total of 37 µM of S100A1 in a buffer containing Ca^2+^, Zn^2+^, or their combinations was loaded into the capillaries and the intrinsic tryptophan fluorescence of the protein was monitored at a temperature range of 20–110 °C. (**A**–**C**) Temperature dependence of the first derivative of I_350_/I_330_ for (**A**) decalcified S100A1 in the presence of 37–296 μM Ca^2+^ (green) or Zn^2+^ (purple), (**B**) Zn^2+^-saturated S100A1 in the presence of 37–296 μM Ca^2+^, (**C**) Ca^2+^-saturated S100A1 in the presence of 37–296 μM Zn^2+^. (**D**) Mid-transition temperatures (T_m_) for S100A1 in presence of different ions, determined from the first derivative of I_350_/I_330_.

Since S100A1 contains only one tryptophan residue, the changes in the fluorescence of the protein reflect only local conformational modifications in the protein structure. Thus, to investigate the impact of zinc and calcium ions on the global protein structure, we examined thermal stability of S100A1 by monitoring the temperature dependence of intrinsic tryptophan fluorescence at 350 nm and 330 nm in the presence of increasing concentrations of Ca^2+^ and Zn^2+^ (Figure 6). Interestingly, although the binding of the first calcium ion in the high-affinity site induced important conformational changes in the local environment of Trp90, it did not affect the thermostability of the protein (Figure 6A,D). In contrast, the binding of the second calcium ion in the medium-affinity site did not impact the Trp90 environment, but did progressively stabilize the S100A1 structure. As for Zn^2+^, its binding to both high- and medium-affinity sites progressively destabilizes S100A1 structure, but this effect was more pronounced for the high-affinity site (Figure 6A,D). The titration of the Zn^2+^-saturated protein by Ca^2+^ does not affect its stability, thereby confirming the absence of Ca^2+^ binding (Figure 6B,D). By contrast, the titration of the Ca^2+^-saturated protein by Zn^2+^ reduces its stability, but no more than to the baseline level, similar to that of the apo-form (~71 °C) (Figure 6C,D). Given that Zn^2+^ binding to high-affinity sites in the EF-hands would result in a more pronounced drop in the temperatures of denaturation (Figure 6D), this effect indicates that Zn^2+^ replaces Ca^2+^ only from low-affinity sites, yielding a 2Zn^2+^:S100A1:2Ca^2+^ form. We also determined the aggregation temperatures of these forms by monitoring light scattering at 350 nm, which yielded very similar results (Figure S2).

## 4. Discussion

This study reports new data regarding the mechanisms of Ca^2+^ and Zn^2+^ binding to S100A1, a crucial multitarget regulatory protein abundant in the heart, nervous system and the retina. Using ESI-MS, we directly demonstrate the existence of the Ca^2+^- and Zn^2+^-containing complexes of S100A1. Indeed, this method is well suited for establishing the stoichiometry of metals binding to proteins [37,38]. Our results indicate that the S100A1 homodimer binds up to 4 calcium ions per monomer, although the most abundant forms contain one or two calcium ions bound to EF-hands, which is in agreement with the available NMR and crystal structures of the protein [35,36]. According to the results of the ITC studies (performed in the presence of TCEP to reduce possible disulfide complexes of the protein [39,40]), S100A1 binds first two calcium ions with submicromolar (K_D_ = 164 nM) and micromolar (K_D_ = 24 μM) affinities. The second constant corresponds to the site located in the N-terminal EF-hand, which is sometimes called the pseudo-EF-hand, due to weak calcium coordination [10]. The additional third and fourth calcium ions are captured outside of the EF-hands, and their binding is non-specific, which is supported by the ITC data. These observations are generally in agreement with the early data obtained using flow dialysis, although in our study the affinity of the best Ca^2+^-binding site is found to be two orders of magnitude higher than in previous assessments [13]. 

In the case of Zn^2+^, we observed a similar stoichiometry, but with stronger binding: the best two sites in each of S100A1 monomers exhibited nanomolar affinities (K_D_ ~ 4 and 770 nM). These findings differ drastically from the data obtained in previous work, where the dissociation constants of Zn^2+^-S100A1 complexes were estimated to be > 10 μM [13]. This discrepancy may be related to the thionylation of S100A1 by β-mercaptoethanol, which is known to alter the Ca^2+^-sensitivity of the protein and may affect its affinity to Zn^2+^ [39]. A more recent study employing ITC revealed only one Zn^2+^-binding site in S100A1, with K_D_ of 3.5 μM [41]. We suppose that the failure in determining the second high-affinity Zn^2+^-binding site was due to the insufficient decalcification of S100A1, since, according to our observations, its incubation with Chelex-100 resin (the method used for the decalcification of S-100 proteins in [41]) does not completely remove calcium from the protein. 

Interestingly, our ITC and nanoDSF experiments demonstrate that Ca^2+^ and Zn^2+^ compete for the high-affinity binding sites in S100A1, thereby confirming that both metals bind to EF-hands. Moreover, the highest affinities were registered in the case of the first Zn^2+^-binding site (N-terminal EF-hand) and the first Ca^2+^-binding site (C-terminal EF-hand), pointing to the existence of a mixed Zn^2+^:S100A1:Ca^2+^ form of the protein. Indeed, it dominates in MS spectra, along with Ca^2+^-loaded and Zn^2+^-loaded conformers. The difference in the Ca^2+^/Zn^2+^ affinities of the EF-hand sites can be explained by comparing the coordination spheres of these cations based on NMR data and the results of the QM/MM simulations performed in our study [36]. The key dissimilarity between these coordination spheres is the number of charged acidic residues, namely one carboxyl in the N-terminal EF-hand and three carboxyls in the C-terminal EF-hand. The binding of calcium in the N-terminal EF-hand is less favorable due to suboptimal coordination by backbone oxygen atoms and the low compensation of positive charge of the metal in the absence of negatively charged ligands. In the case of zinc binding, one of the backbone oxygens is replaced with a water molecule (stabilized by this oxygen and the carboxyl group of E32) which, together with the oxygen from the S19 side group, ensures octahedral coordination, making Zn^2+^ tightly bound through the maximal number of chelators (coordination number of Zn^2+^ in proteins is 4–6) [42]. In the C-terminal EF-hand, Zn^2+^ is coordinated in a similar fashion by capturing all five chelators and the water molecule. In this site, Ca^2+^ also binds in optimal coordination by these six chelators, including three carboxyls. Notably, the principle difference between Zn^2+^ and Ca^2+^ is the more hindered dehydration of Ca^2+^ when chelating with amide or carbonyl oxygen atoms (analogs of backbone oxygen atoms) as compared to chelation by carboxyl oxygen atoms [43,44]. Overall, Zn^2+^ binding is by default more favorable in the N-terminal EF-hand of S100A1, whereas Ca^2+^ binding is slightly more favorable in the C-terminal EF-hand, although in the latter case the affinities are comparable. 

These new data significantly update our current understanding of the composition and behavior of the S100A1 metal-bound forms under physiological conditions. It is known that the extracellular concentration of Ca^2+^ is 1.5–2 mM, whereas the intracellular levels of this metal range from 50 to 100 nM in the mammalian brain and up to 600 nM in the retina [45,46]. The internal concentration of Zn^2+^ in the neurons is at a low picomolar level, whereas its external concentration in some cases may reach up to 300 μM, such as in the synaptic clefts of glycinergic neurons [47]. In addition, the level of free Zn^2+^ may aberrantly increase outside the synapses and provide neurotoxicity in neurodegenerative diseases, such as AD and glaucoma [18,48]. Given our findings regarding the much higher Ca^2+^-sensitivity of S100A1, one can suspect that the protein could actually be sensitive to Ca^2+^ signals within a cell, even in the absence of its targets. Furthermore, the nanomolar affinity of S100A1 to Zn^2+^ revealed in our study indicates that it could function as a Zn^2+^-signaling protein by exchanging the so-called loosely-bound zinc (K_D_ ~ 10^−8^–10^−6^ M^−1^) with other proteins in response to stimuli [19,49]. Thus, apo, Ca^2+^-loaded and Zn^2+^-loaded S100A1 seem to be the most probable states of the protein within a cell. In turn, being secreted into the extracellular space, S100A1 might adopt a Ca^2+^-loaded conformation, as the calcium concentration outside the cell is 2 orders of magnitude higher than the K_D_ for the medium-affinity Ca^2+^-binding site of the protein [50,51]. Yet, even Ca^2+^-loaded S100A1 can respond to increased free Zn^2+^ by forming 2Zn^2+^:S100A1:2Ca^2+^ and can thereby participate in the pathophysiological processes leading to neurodegeneration. 

How would high-affinity Ca^2+^ and Zn^2+^ binding reflect on the stability and conformational states of S100A1? To answer this question, we first performed DLS studies, as this method allows the monitoring of the effect of cations on the global structural integrity and multimerization of proteins [52]. Our results demonstrate that, at physiological temperature (37 °C), S100A1 remains as a dimer regardless of the Ca^2+^ or Zn^2+^ concentrations although this dimer adopts a more compact conformation at high (10 mM) Zn^2+^ levels. However, according to intrinsic fluorescence (nanoDSF) studies, Ca^2+^-bound and Zn^2+^-bound S100A1 already adopt different conformations at the binding of the first metal ion per monomer. The most pronounced change was observed upon binding of the first two ions i.e., in the high-affinity EF-hand sites, in agreement with previous estimations [13]. Notably, the binding of Ca^2+^ increases the thermal stability of S100A1, whereas Zn^2+^ significantly decreases the temperatures of its denaturation and aggregation. Thus, the most probable physiological forms of S100A1, namely apo, Ca^2+^-loaded and Zn^2+^-loaded, differ in overall structure, conformational flexibility and environmental resistance, and therefore may display different physiological activities. The validation and structural characterization of the revealed additional S100A1 forms, such as Zn^2+^:S100A1:Ca^2+^ and 2Zn^2+^:S100A1:2Ca^2+^, requires further studies.

The discovery in this study of the new physiologically relevant Zn^2+^-loaded and Zn^2+^(Ca^2+^)-bound conformers of S100A1 suggests that Zn^2+^-dependent regulation could be a novel aspect in its function. Thus, Zn^2+^ can modulate both the Ca^2+^-dependent and Ca^2+^-independent activities of S100A1, which is known to play important roles in cells not only at high Ca^2+^, but also under resting Ca^2+^ conditions [53,54]. Zinc binding was shown to have a direct impact on the activity of S100A1. For instance, it enhances S100A1-dependent activation of twitchin kinase, although it is unclear if this effect is mediated by Zn^2+^ binding to Ca^2+^-loaded S100A1, the target protein, or both [55]. Some S100 proteins could function as metallochaperones by releasing Zn^2+^ to be taken up by their target proteins [10]. S100A1 binds p53, as well as a few other Zn^2+^-binding proteins, including tubulin, tau-protein, phosphoglucomutase, and different S100 proteins [56,57,58]. For instance, in the case of tau, the dissociation constant for Zn^2+^ is ~10^−7^ M, which is comparable to those determined for S100A1 in this work, which could theoretically lead to exchange of zinc ions between the two proteins in both directions. By contrast, in the case of tubulin, the constant is significantly lower (K_D_ ~ 10^−5^ M), which means that S100A1 could act as a Zn^2+^ acceptor rather than a donor when forming a complex with tubulin [56,59]. This suggestion is supported by the stabilizing effect of S100A1 on microtubules in the presence of Zn^2+^, which otherwise induces their disassembly [60]. 

Notably, our results show that the excessive binding of Zn^2+^ destabilizes S100A1 and increases its susceptibility to aggregation. Zinc-induced protein aggregation is a hallmark of neurodegenerative diseases such as Alzheimer’s disease and amyotrophic lateral sclerosis [31,61]. Since S100A1 can be secreted in the extracellular space, where high amplitude Zn^2+^ fluctuations are observed, we propose that it can contribute to pathogenesis of Zn^2+^-dependent neurodegenerative diseases such as AD and glaucoma [50,51]. S100A1 is found in the amyloid plaques of AD patients and is known to contribute to plaque formation, neuroinflammation and cell death in AD models [62]. In addition, S100A1, together with amyloid peptides, was detected in the aqueous humor of glaucoma patients [8,9]. S100A1 expression is dysregulated in type I diabetes, which is characterized by altered Zn^2+^ homeostasis [63,64,65]. Furthermore, the upregulation of S100A1 is found in tumors such as melanoma (> 100-fold increase), renal cell carcinoma and ovarian cancer [65,66,67,68]. S100A1 was reported to control the migration and proliferation of cancer cells and its elevated expression is a known marker of tumor malignancy [65]. As cancer cells are characterized by redox imbalance and oxidative stress is a major source of excess Zn^2+^ in cells (the oxidation of sulfhydryl groups in Zn^2+^-buffer proteins leads to massive Zn^2+^ release), S100A1 can predominantly act as a Zn^2+^-saturated conformer in cancer cells [69]. This should be considered while searching for approaches to suppressing the pathological function of S100A1. Indeed, targeting the Zn^2+^-dependent metabolic and signaling pathways in tumors with specific chelators is regarded as a prospective strategy, because it could help sensitize cancer cells to established chemotherapy drugs [70,71]. 

In conclusion, this work demonstrates, for the first time, the high affinity binding of Ca^2+^ and Zn^2+^ by a S100A1 homodimer, which results in the formation of physiologically relevant, Ca^2+^/Zn^2+^-bound conformers of this protein that are structurally and, potentially, functionally different. New data acquired by direct analysis of the complex by ESI-MS mass-spectrometry and monitoring of the interactions by ITC shows that two calcium and two zinc ions compete for the same sites within the S100A1 structure. In correspondence with the results of ITC, novel Zn^2+^-binding sites within the EF-hands of the protein were predicted using QM/MM molecular modeling. Furthermore, two additional zinc ions can bind outside of the EF-hands, yielding the 4Zn^2+^:S100A1 or 2Zn^2+^:S100A1:2Ca^2+^ forms of the protein, which possess somewhat compromised structural stability. Although further studies are required for more detailed examination of the functional properties of the newly revealed S100A1 forms, the results of this study generally contribute to the understanding of Ca^2+^/Zn^2+^ interplay in cell signaling in health and disease.

## Figures and Tables

**Table 1:** Thermodynamic parameters of Ca^2+^ and Zn^2+^ binding to S100A1.

Ion	N^1^	K^1^_a_, M^−1^	K^1^_D_, M	∆H^1^, kcal M^−1^	∆S^1^, cal M^−1^ K^−1^	N^2^	K^2^_a_, M^−1^	K^1^_D_, M	∆H^2^, kcal M^−1^	∆S^2^, cal M^−1^ K^−1^
Ca^2+^	0.9	6.1 ± 2.1 × 10^6^	1.6 ± 0.6× 10^−7^	−16.9 ± 0.2	−23.3	1.0	4.2 ± 0.6 × 10^4^	2.4 ± 0.3× 10^−5^	4.0 ± 0.8	34.1
Zn^2+^	0.9	2.6 ± 0.7 × 10^8^	3.8 ± 1.0× 10^−9^	−13.2 ± 0.2	−4.2	0.9	1.3 ± 0.4 × 10^6^	7.7 ± 2.4× 10^−7^	3.3 ± 0.4	38.5
Ca^2+^ in Zn^2+^		no binding								
Zn^2+^ in Ca^2+^		no binding								

All parameters were determined using the “two sets of sites” model.

**Table 2:** Secondary structure fractions estimated from CD spectra for apo, Ca^2+^-loaded (1 mM CaCl_2_), and Zn^2+^-loaded (1 mM ZnCl_2_) forms of S100A1.

Protein State	α-Helices, %	β-Structure, %	Turns, %	Unordered Structure, %
apo	70.58 ± 0.56	5.17 ± 0.09	8.01 ± 0.40	16.02 ± 0.25
Ca^2+^-loaded	68.05 ± 0.56	4.05 ± 0.09	9.28 ± 0.40	18.65 ± 0.25
Zn^2+^-loaded	65.10 ± 0.56	4.13 ± 0.09	11.17 ± 0.40	19.50 ± 0.25

## Supplementary Materials

The following are available online at https://www.mdpi.com/article/10.3390/biom11121823/s1, Figure S1: Far-UV CD spectra of S100A1; Figure S2: S100A1 aggregation temperatures.

## Abbreviations

ESI-MS—electrospray mass spectrometry, ITC—isothermal titration calorimetry, DLS—dynamic light scattering, nanoDSF—nano-format of differential scanning fluorimetry, QM/MM—quantum mechanics/molecular mechanics.
