# Supplementary material for: Mechanism of Zn2+ and Ca2+ Binding to Human S100A1
Source: Biomolecules. 2021 Dec 3;11(12):1823. doi: 10.3390/biom11121823 (PMC8699212; doi:10.3390/biom11121823)
Supplement: Supplementary file 1 [file biomolecules-11-01823-s001.zip › biomolecules-1452864-supplementary.pdf]

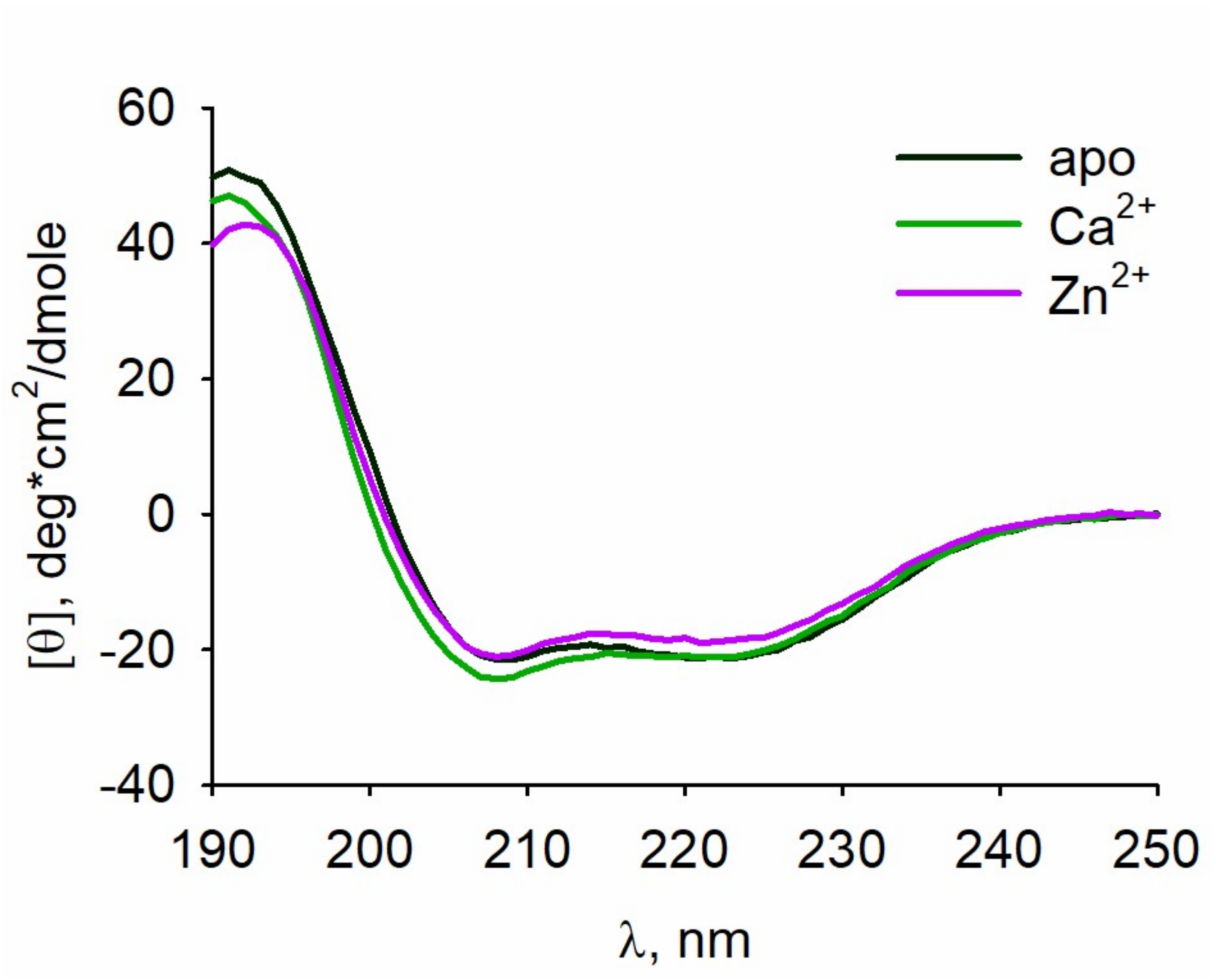

Figure S1. Far-UV CD spectra of 37 μM apo (black line), Ca^{2+}-loaded (1 mM $CaCl_2$, solid green line) and Zn^{2+}-loaded (1 mM $ZnCl_2$, solid purple line) S100A1 at 37 °C.

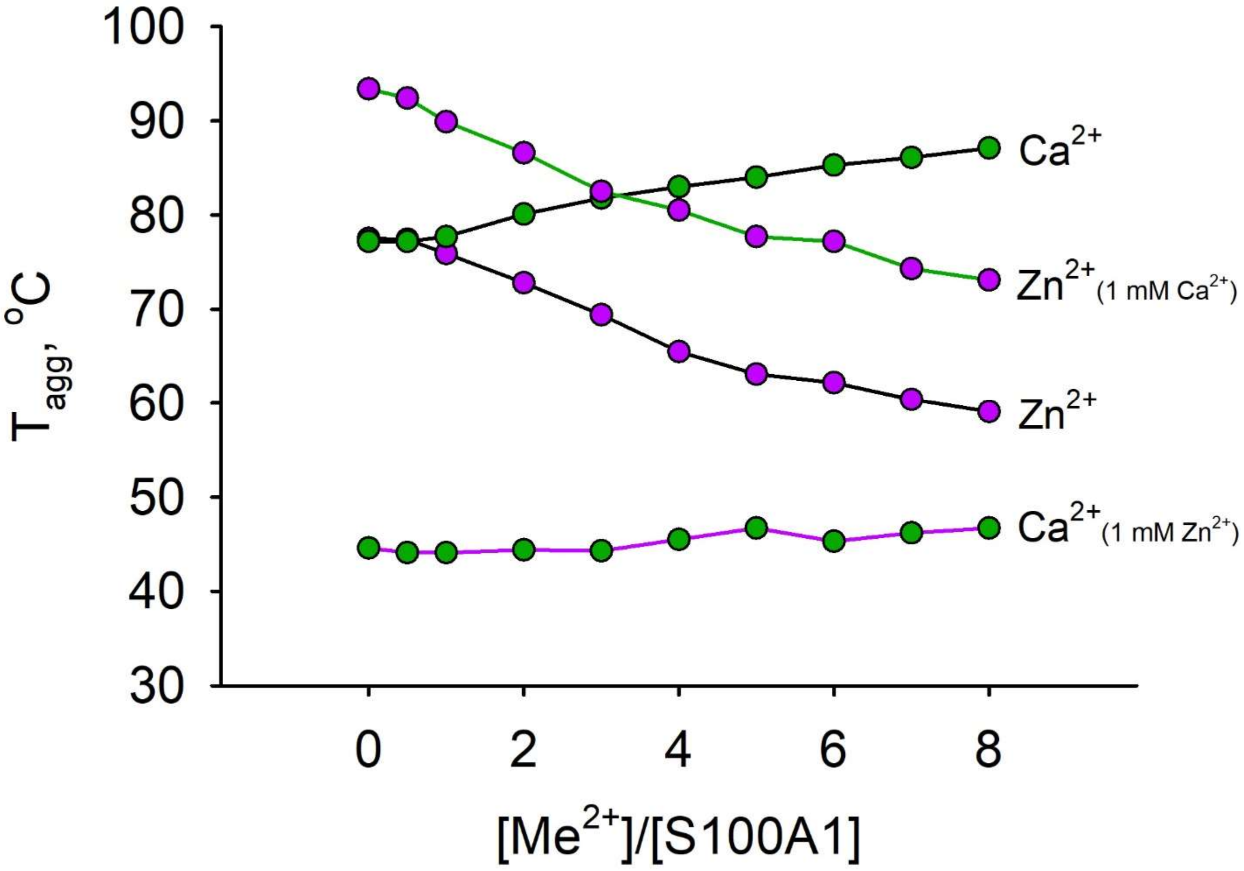

Figure S2. Effect of Ca^{2+} and Zn^{2+} on aggregation of S100A1. 37 μM of S100A1 in a buffer containing Ca^{2+}, or Zn^{2+}, or their combinations was loaded into capillaries and light scattering at 350 nm was monitored at temperature range 20–110 °C. Aggregation temperatures (T_{agg}) for S100A1 in presence of different ions were determined from the first derivative of light scattering temperature dependance.
